# Propagation and Sequencing of African Swine Fever Virus on Porcine-Derived Buffy Coat Fraction Cells

**DOI:** 10.1155/tbed/1160908

**Published:** 2025-07-30

**Authors:** Jessica Mason, Mariceny Zurita, Lauren Martignette, Janine Simmons, John G. Neilan, Michael Puckette

**Affiliations:** ^1^Plum Island Animal Disease Center, SAIC, P.O. Box 848 11944, Greenport, New York, USA; ^2^U.S. Department of Homeland Security Science and Technology Directorate, Plum Island Animal Disease Center, P.O. Box 848, Greenport 11944, New York, USA; ^3^U.S. Department of Agriculture, National Bio and Agro-Defense Facility, Biologics Development Module, P.O. Box 1807, Manhattan 66502, Kansas, USA

**Keywords:** African swine fever virus, buffy coat, hemadsorption, primary cells, sequencing, swine, virus isolation

## Abstract

African swine fever (ASF) has emerged as a preeminent threat to worldwide pork production. Research and diagnostics of ASF virus (ASFV) is dependent upon culturing virus in primary cells, such as peripheral blood macrophages (PBMC) derived from swine blood, or pulmonary alveolar macrophages (PAM) extracted from swine lungs. The methodologies for production of these cells can be laborious, requiring significant investment in vivarium, personnel, and technical resources. As an alternative, the buffy coat cell fraction from blood contains a mixture of cell types, including undifferentiated monocytes that can be easily isolated by centrifugation. Herein, we culture buffy coat cells in macrophage (M∅) base media, containing L929 conditioned media to induce monocyte differentiation and enhance sensitivity to ASFV. Culturing the buffy coat cell fraction in M∅ base media enhanced the abundance of rosettes and number of detectable ASFV genome copies relative to buffy coat cells grown without L929 conditioned media. Buffy coat fraction cells were used to propagate ASFV directly from blood of infected swine and subsequent sequencing of extracted viral DNA yielded full genome coverage and identification of point mutations. This work demonstrated that growing ASFV in cells of the buffy coat fraction for pig blood was an effective alternative to using the traditionally isolated primary cell types for ASFV propagation, isolation, and sequencing.

## 1. Introduction

African swine fever (ASF) is a highly contagious viral disease of swine caused by the ASF virus (ASFV), the only member of the DNA virus family *Asfarviridae*. First described in 1921 [1], ASF is endemic in wild *Suidae* populations of Africa. In 2007, an ASF outbreak in the Republic of Georgia spread throughout Eurasia and the island of Hispaniola, resulting in substantial worldwide swine losses [2–8]. While ASF is spread through arthropod feeding in endemic regions, the current outbreak has largely spread through the transport of contaminated material and pork products [9–12].

Full genome sequencing has emerged as an important tool in the study of ASF [13, 14]. Sequencing of multiple ASFV genomes in the Dominican Republic identified the geographic lineage of the introduced virus as well as genetic clusters within Hispaniola [13]. While sequencing of full ASFV genomes has been reported from infected spleen tissue [15], the quantity and quality of samples submitted from the field can vary widely. Therefore, efficiently culturing ASFV from field samples is required to supply enough quality viral DNA for sequencing.

Identification of immortalized cell lines capable of propagating ASFV are a recent development [16, 17] with none being widely available, making adoption and utilization difficult. ASFV research remains dependent on primary cells for virus propagation and amplification, typically either peripheral blood macrophages (PBMC) derived from swine blood [18–20] or pulmonary alveolar macrophages (PAM) extracted from swine lungs [21, 22]. Both cell types are highly effective in culturing ASFV, but require significant investment of vivarium, personnel, and technical resources causing limitations on availability. In addition to PBMCs and PAMs other sources of monocyte derived macrophages have been utilized for culturing ASFV including bone marrow [22–24].

Utilization of PBMCs requires large volumes of blood, typically 500 mL to 2 L, and cell separation gradients to isolate non-differentiated cells from swine blood followed by the addition of stimulating factors to induce terminal differentiation [25]. Blood is typically obtained from either a herd of blood donors or through a terminal bleed. Isolation of PAMs requires harvesting and repeated washing of lungs, typically from piglets prior to stimulation of cell differentiation. The need to use pigs specifically for these functions can serve as an impediment for ASFV research and diagnosis in areas lacking the required resources.

Cells from the buffy coat fraction contain both terminally differentiated cells, such as macrophages, and undifferentiated precursors. Isolation of buffy coat fraction cells can be performed with small volumes of blood through centrifugation. Despite buffy coat cells being previously identified as susceptible to infection by ASFV [24, 26], there is little published information on the application of buffy coat cells for propagation of ASFV. None describe buffy coat fraction cells cultured in media containing stimulating factors, such as that produced and secreted by the L929 cell line [27]. In this report we evaluate buffy coat cells extracted from pig blood under multiple conditions for detection, propagation, and sequencing of ASFV.

## 2. Materials and Methods

### 2.1. Culturing of L929 Cells and Harvest of Media Containing GM-CSF

The NCTC clone 929 (L929) cell line (ATCC, CCL-1) is of murine origin and secretes GM-CSF into culture media capable of inducing differentiation of swine monocytes, [18, 20, 28, 29]. To harvest, L929 cells are cultured in growth media (RPMI-1640 with Hepes/L-glutamine, 10% gamma-irradiated fetal bovine serum [FBS], and 1% antibiotic/antimycotic) until fully confluent. Upon confluency, supernatant was harvested and centrifuged at 7440 × *g* for 10 min at room temperature followed by filtration through a 0.22 μm filter and stored at −20°C.

### 2.2. Extraction of Buffy Coat Cells From Swine Blood

Swine blood was obtained in either 4 mL EDTA tubes, 10 mL heparin tubes, or 2 L of heparin-treated blood. Heparin-treated blood was aliquoted into conical tubes at volumes of 5, 15, or 50 mL and centrifuged at 1700 × *g* for 10 min upon receipt. To evaluate blood storage conditions, blood was stored in tubes overnight at 4°C prior to centrifugation. Buffy coat cells appear as a white layer between the plasma and red blood cell fractions, were harvested by pipetting. To lyse residual red blood cells, the buffy coat cell fraction (≈ 2 mL) was transferred to a 15 mL conical tube followed by the addition of 2 mL ammonium chloride solution (STEMCELL, 07850), and incubated on ice for 5 min with gentle swirling every 2 min. After incubation, 10 mL of wash media (1x DMEM, 1% antibiotic/antimycotic) was added to stop lysis followed by centrifugation at 1700 × *g* for 10 min at 4°C. To further reduce the amount of red blood cells, the cell pellet was resuspended in 1 mL of ammonium chloride solution and incubated on ice for 5 min with gentle swirling every 2 min. Wash media, 10 mL, was added to inactivate blood cell lysis, and cells were pelleted by centrifugation at 1700 × *g* for 10 min at 4°C. The cell pellet was resuspended in 10 mL of wash media for a final wash, and cells pelleted by centrifugation at 1700 × *g* for 10 min at 4°C. Following the removal of supernatant, the cell pellet was resuspended in either M∅ base media (49% RPMI, 30% L929 conditioned media, 20% gamma-irradiated fetal bovine serum (FBS), 1% antibiotic/antimycotic, and 0.1% gentamicin) to induce differentiation or M∅ mimic media (76% RPMI, 23% FBS, 1% antibiotic/antimycotic, and 0.1% gentamicin) for comparison with differentiated cells or for counting prior to cryopreservation.

Resuspended cells were counted in a Nucleocounter (Chemometec) and plated on 96- or 6-well Corning Primaria Culture Plates, cell density 2.5 × 10^5^ cells/well and 8 × 10^6^ cells/well, respectively. The Nucleocounter, or similar instrument, is critical as only nucleated cells are counted to quantify the cell density independent of residual red blood cells. Cells were incubated in either 100 μL or 3 mL of M∅ base media per well for 96- and 6-well plates, respectively, at 37°C with 5% CO_2_ for 24 h. After incubation, nonattached cells were removed by washing with either 250 μL or 4 mL of M∅ base media per well for 96- and 6-well plates, respectively. After the wash was removed, 100 μL or 3 mL of fresh M∅ base media was added per well for 96- and 6-well plates, respectively, prior to infection with ASFV.

#### 2.2.1. Cryopreservation of Buffy Coat Cells

For cryopreservation of buffy coat cells, cells were centrifuged and resuspended in cryopreservation media (90% FBS, 10% dimethyl sulfoxide [DMSO]) in 1 mL aliquots at a cell density of 1 × 10^6^ cells per 1 mL. Cells were placed in a Mr. Frosty freezing container (Thermo Fisher) and stored overnight at −80°C prior to storage at −150°C.

For recovery of cryopreserved cells, tubes were rapidly thawed in a 37°C incubator, and the contents transferred to a 15 mL conical tube. Freezing media was diluted by adding 10 mL of M∅ base media, and cells were pelleted by centrifugation at 1700 × *g* for 10 min. The supernatant was removed, and cells resuspended in M∅ base media to produce desired cell concentrations.

### 2.3. Infection of Buffy Coat Cells With AFSV

#### 2.3.1. Infection of Cells in M∅ Base Media and M∅ Mimic Media

Buffy coats were resuspended in either M∅ base media or M∅ mimic media and plated on 6-well plates. Three wells of cells from each swine were used with both media types. Wells were infected using 0.5 μL of ASFV isolate Georgia 2007 stock virus, 10^6.8^ TCID_50_/mL, an approximate MOI of 4 × 10^−4^. Infected cells were incubated at 37°C with 5% CO_2_. At 3 days postinfection (dpi) one well for each sample received 80 μL of diluted swine blood (25% red blood cells in 1 x dPBS) to evaluate rosette formation 24 h postapplication.

Supernatant from infected cells was harvested at 5, 7, 10, and 14 dpi and analyzed by real time PCR on an Applied Biosystems 7500 (Thermo fisher) using primers and protocol as previously published [30]. Real Time PCR reactions were performed in triplicate for each sample, and the *C*_T_ values were averaged for the three pigs.

#### 2.3.2. Screening of AFSV Isolates on Buffy Coat Cells

Five historical ASFV isolates, Brazil '78, Haiti '79, Lisbon '60, Tengani ‘62, and Killean III, were obtained from the United States Department of Agriculture (USDA) Animal and Plant Health Inspection Service (APHIS) Foreign Animal Disease Diagnostic Laboratory (Plum Island Animal Disease Center) and propagated for one passage on PAM (RTI LLC) to create virus working stocks. Buffy coat cells were plated on 96-well plates in M∅ base media, as described in Section 2.2. For infection, 110 μL per well of M∅ base media containing a 2.75 × 10^−3^ dilution of virus stocks was utilized for all isolates. Red blood cells were applied 3 dpi, and wells were evaluated for rosettes at 4 dpi.

#### 2.3.3. Infection With Blood From Experimentally Infected Swine

Blood samples from swine experimentally infected with ASFV Georgia 2007 as part of ongoing studies were generously provided by the USDA Agricultural Research Service Foreign Animal Disease Research Unit and the USDA APHIS (Plum Island Animal Disease Center). Blood samples were aliquoted and stored at 4°C.

For real time PCR of ASFV positive blood, blood from individual pigs experimentally infected with ASFV, was serially diluted 10-fold with M∅ base media. Real time PCR for detection of ASFV was performed as previously described [30].

To evaluate replication over time at several dilutions of virus stock or blood, both an ASFV isolate Georgia 2007 stock virus, 10^6.8^ TCID50/mL, and ASFV-positive blood was diluted to 1% concentration in M∅ base media. A serial dilution, 10^−2^ to 10^−12^, was performed in duplicate on 96-well plates with two separate buffy coat cell stocks. Samples of cell culture media were collected at 1, 5, 7, 11, and 14 dpi, and real time PCR was performed using four replicates from each sample on an Applied Biosystems 7500 (Thermo fisher) using previously published primers and protocol [30]. Results were averaged to calculate a final *C*_T_ value.

To examine infectivity of ASFV using stored blood samples, ASFV-infected blood was diluted to 1% in M∅ base media, serially diluted 10-fold, and applied to 96-well plates containing cells. Cells were incubated at 37°C for 24 h with 5% CO_2_. Media was removed, and 100 μL of M∅ base media was applied to the cells incubated for two additional days. At 3 dpi, 2.5 μL of diluted swine blood (25% red blood cells in 1 x dPBS) was applied to each well, and the presence of rosettes was scored at 4 and 6 dpi.

#### 2.3.4. Propagation of ASFV From Infected Blood for Sequencing

For sequencing, 10 μL of blood from an unvaccinated swine infected with the Georgia 2007 ASFV isolate as part of an unrelated vaccine challenge study was diluted in 1 mL of M∅ base media to make a working stock from which 100 μL was added to either two wells of a 6-well plate for each sample or a T-50 flask. Buffy coat cells were plated at densities of 8.0 × 10^6^ per well for 6-well plates and 4.2 × 10^7^ cells per T-50 flask. Infected cultures were incubated at 37°C with 5% CO_2_ for 7 days after which cells were removed by pipetting and transferred to a 15 mL conical tube followed by centrifugation at 1847 × *g* for 15 min. The pellet was resuspended in 850 μL of NTE buffer (10 mM Tris [pH 7.6], 5 mM EDTA, 100 mM NaCl) followed by addition of 50 μL of 10% sodium dodecyl sulfate and 100 μL of protease K (10 mg/mL). Samples were mixed by inversion five times and incubated at 37°C with 5% CO_2_ for 1.5 h. Following incubation, 250 μL of 5 M NaCl was added, samples were mixed by inversion and stored at −20°C overnight. The following day samples were thawed and transferred to 1.5 mL tubes for centrifugation at 14,462 × *g* for 30 min at 4°C. The supernatant containing DNA was transferred to a new tube and an equal volume of a 25:24:1 mixture of phenol:chloroform:isoamyl alcohol was added followed by inversion 10 times prior to centrifugation at 14,500 × *g* for 3 min. The top layer was transferred to a new tube followed by the repeated addition of an equal volume of 25:24:1 mixture of phenol:chloroform:isoamyl alcohol. Centrifugation at 14,500 × *g* for 3 min was repeated with the harvested layer. The top layer was harvested and 5 M sodium acetate, pH 5, and 100% ethanol, equivalent to 0.1 and 2.5 times the volume of retained fractions, respectively, were added followed by incubation for 30 min at −70°C. Samples were centrifuged at 7378 × *g* for 30 min to precipitate the DNA. DNA pellets were washed with 70% ethanol and centrifuged at 7378 × *g* for 5 min and air dried prior to suspension in 100 μL of nuclease free water.

Extracted DNA was evaluated by real time PCR, as described in Section 2.3.1 to assess the presence of ASFV DNA, and the ASFV DNA concentration was determined by Qubit dsDNA assay. Sequencing was performed by the GARA Center for AFSV Genomics (asfvgenomics.com) at Plum Island Animal Disease Center utilizing the standardized sequencing pipeline described previously [31]. Full genome sequencing was performed on an Illumina NextSeq 500, and the reads were aligned to the ASFV Georgia 2007 reference sequence (Genbank ID: LR743116) for analysis.

## 3. Results

### 3.1. Replication of ASFV in Cells of the Buffy Coat Fraction is Enhanced by Inclusion of L929 Media

**Figure 1:**
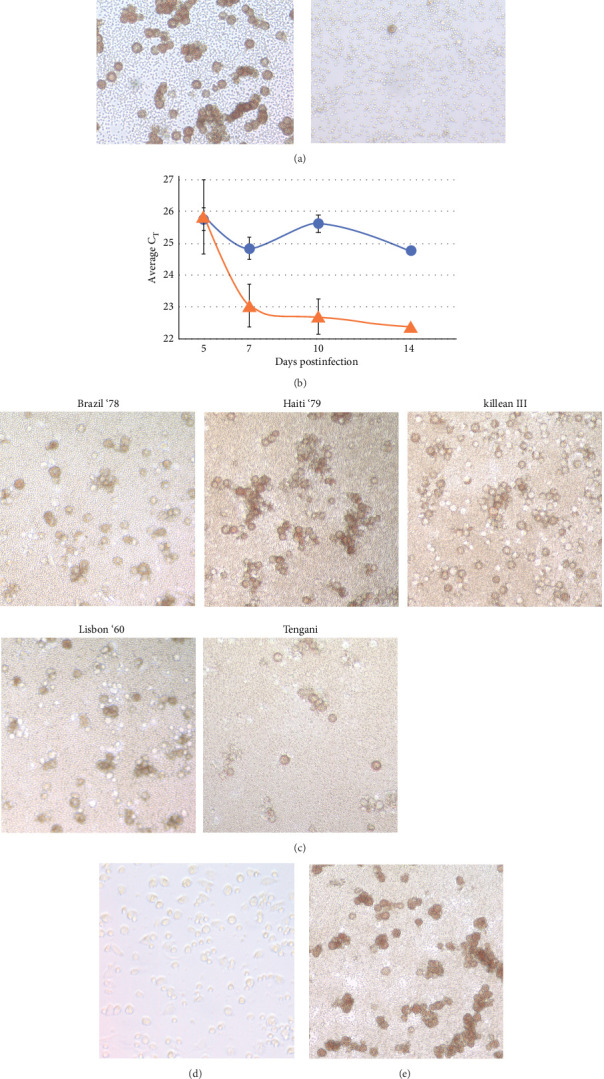
(A) Inclusion of L929 cell culture media in M∅ base media increased the abundance of rosettes compared to those observed in M∅ mimic media. (B) A statistically significant decrease, *p* ≤ 0.001 as determined by a Student's *T*-test, in average *C*_т_ values was observed at 7-, 10-, and 14-days postinfection in M∅ base media (orange triangles) when compared with M∅ mimic media (blue circles) demonstrating greater viral genome numbers in M∅ base media samples. (C) Rosette formation on buffy coat cells for six ASFV isolates: Brazil '78, Haiti '79, Killean III, Lisbon '60, and Tengani. (D) Buffy coat cells cryopreserved and stored at −150°C for 12 days retained viability and (E) the ability to produce rosettes upon infection.

Buffy coat cells were cultured in either M∅ base media or M∅ mimic media followed by infection with the ASFV Georgia 2007 isolate. Rosettes were observed at 4 dpi, 1 day postapplication of red blood cells. While rosettes were observed in cells cultured in both M∅ base media and M∅ mimic media, they were more abundant in M∅ base media (Figure 1A).

Rosette formation is an indicator of ASFV protein expression. To confirm viral genome replication, cell culture supernatant was tested by real time PCR at 5, 7, 10, and 14 dpi (Figure 1B). Supernatant from infected cells in M∅ base media demonstrated a decreasing average *C*_т_ value from 25.76 (± 1.23) to 22.37 (± 0.09) between 5 and 14 dpi, respectively, indicative of an increasing amount of ASFV genomes. Supernatant from infected cells in M∅ mimic media contained only a minimal decrease in the *C*_т_ values, from 25.75 (± 0.35) to 24.81 (± 0.15) during the same time points. Data for all real time PCR replicates used in Figure 1B can be found in Supporting Information.

To determine if cells of the buffy coat fraction in M∅ base media could be infected with lower virus concentrations, stock virus was diluted, and the growth media was evaluated by real time PCR at 1, 5, 7, and 14 dpi (Table 1). No wells at dilutions between 10^−9^ and 10^−11^ yielded positive real time PCR results. All dilutions except 10^−8^ demonstrated a decrease in average *C*_т_ values over time, indicative of ASFV replication. ASFV grew in all wells over the time course down to a 10^−5^ dilution with positive wells present in 10^−6^ through 10^−7^ dilutions. At 10^−8^ a single well was PCR positive at 1 dpi, but no wells were positive at later time points, suggestive of the presence of ASFV DNA but not viable virus. Data for all real time PCR replicates used in Table 1 can be found in Supporting Information.

Five additional strains of ASFV: Brazil '78, Haiti '79, Lisbon '60, Tengani, and Killean III, representing different genotypes then the Georgia isolate, were tested on buffy coat cells producing abundant rosettes, indicating the potential application of buffy coat cells cultured in M∅ base media for multiple ASFV genotypes (Figure 1C). ASFV strains Lisbon ‘60 and Killean III were evaluated by real time PCR to confirm viral genome replication. Both demonstrated statistically significant decreases in *C*_т_ values, *p*  < 0.001, indicative of increases in ASFV genomes between 5 and 7 dpi. Lisbon ‘60 *C*_т_ values decreased from 23.3 (± 0.5) to 21.0 (± 0.4) and Killean III *C*_т_ values decreased from 26.5 (± 0.4) to 23.3 (± 0.2), confirming ASFV genome replication.

Cryopreservation of isolated cells avoids perpetual maintenance of swine blood donors. Buffy coat cells were cryopreserved at −150°C for 12 days, thawed, and evaluated for retention of the ability to propagate ASFV. Cells were recovered and attached to the plate surface (Figure 1D). Following infection with ASFV and addition of diluted red blood cells, rosettes were observed at 2 dpi (Figure 1E), demonstrating retention of ASFV permissiveness. Cells from the same batch tested at 4 months postcryopreservation also retained ASFV permissiveness, data not shown.

### 3.2. Effect of Blood Collection and Processing Methodology

**Figure 2:**
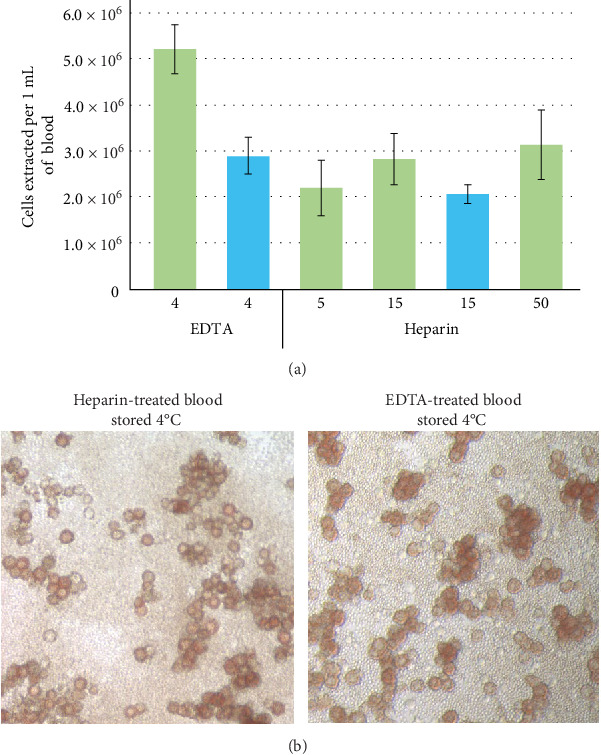
(A) Cells extracted per 1 mL of blood using 4 mL of EDTA-treated blood or 5, 15, or 50 mL of heparin-treated blood. Blood was either processed upon receipt (green) or stored at 4°C overnight (blue). (B) Buffy coat cells retained the ability to produce rosettes upon ASFV infection regardless of anticoagulant used or overnight storage at 4°C.

Two common factors affecting blood collection, availability of anticoagulant tubes and storage of blood prior to processing, were evaluated. Blood from a single source was tested for changes in the number of harvested nucleated cells and ASFV permissiveness in the presence of either EDTA or heparin as anticoagulants. A statistically significant drop, *p*  < 0.001, in buffy coat cell yield per volume of blood was observed when comparing heparin to EDTA as an anticoagulant (Figure 2A). The statistical significance of this difference is lessened when comparing each volume of heparin individually to EDTA, *p*  < 0.005 for 5 and 15 mL volumes and *p*  < 0.02 for 50 mL. The difference in the density of cells extracted among the three different volumes of heparin-treated blood, 5, 15, and 50 mL, did not demonstrate significance by a Student's *T*-test or ANOVA (*p*-value = 0.25). Storage of blood at 4°C reduced the average cell yield per volume of blood for both 4 mL EDTA and 15 mL heparin aliquots (Figure 2A). This difference was significant by ANOVA analysis for EDTA treated blood (*p*-value = 0.004) but not heparin-treated blood (*p*-value = 0.7). Data for individual replicates of cell counts used in Figure 2A can be found in Supporting Information.

Despite alterations in cell yields, blood treated with either anticoagulant and stored at 4°C retained the ability to propagate ASFV and produce rosettes (Figure 2B). To determine if either treatment altered sensitivity of the cells to ASFV, cells were infected with 10^−3^ to 10^−6^ dilutions of stock virus at a concentration of 10^6.8^ TCID_50_/mL. All cells demonstrated similar sensitivity across the tested dilutions with ≥ 50% of wells infected at 10^−5^ for all samples except 4°C stored EDTA blood which demonstrated ≥ 50% of wells infected at the 10^−6^ dilution. These results were utilized to calculate a TCID_50_/mL for virus on cells extracted from blood treated with EDTA or heparin and processed either fresh or stored at 4°C for 24 h. For blood samples processed fresh a titer of 10^6.5^ TCID_50_/mL was obtained on EDTA treated blood while a titer of 10^6.7^ TCID_50_/mL was obtained from heparin treated blood. For blood stored at 4°C for 24 h a titer of 10^7.1^ TCID_50_/mL was obtained on EDTA treated blood while a titer of 10^6.6^ TCID_50_/mL was obtained from heparin treated blood.

### 3.3. Use of Buffy Coat Cells With Blood From ASFV-Infected Swine

During an ASF outbreak a critical need is the ability to culture ASFV from biological samples, such as blood, for isolation, sequencing, and identification of new and emerging isolates. To determine if cells from the buffy coat fraction could propagate virus from blood directly, we compared the replication of ASFV in dilutions of blood from experimentally infected swine on buffy coat fraction cells (Table 2).

Diluted ASFV-positive blood was tested by real time PCR without culturing on primary cells, yielding positive results up to a 10^−6^ dilution (Table 2). To evaluate culturing on cells of the buffy coat fraction, dilutions of the ASFV-positive blood sample were tested. Positive *C*_т_ values were measured in all wells up to a 10^−7^ dilution (Table 2). Higher dilutions of 10^−8^ and 10^−10^ also generated positive wells, 3 and 1, respectively, with progressively lower *C*_т_ values over time. Data for all real time PCR replicates used in Table 2 can be found in Supporting Information.

To assess if the results from a single pig translated more broadly, blood from eight experimentally infected pigs was applied to cell cultures at dilutions between 10^−2^ and 10^−7^. All seven samples produced rosettes in wells at multiple dilutions. The highest positive dilution was a single well at a 10^−7^ from animal 42 (Table 3). All seven samples had been stored at 4°C for over a year, the oldest exceeding 1200 days at 4°C, demonstrating the stability of ASFV over time under these storage conditions.

### 3.4. Viral Genome Sequencing of Virus Cultured on Buffy Coat Cells

To produce ASFV DNA for sequencing, five blood samples were inoculated into either 6-well plates or a T-50 flask of buffy coat cells and cultured in M∅ base media. Viral DNA was extracted using a modified Hirt method and confirmed by real time PCR followed by quantification of double stranded DNA (Table 4). All samples produced viral DNA as confirmed by real time PCR with *C*_T_ scores ranging from 17.2 to 29.3 with estimated concentrations of extracted dsDNA ranging from 6.19 to 36.6 ng/μL (Table 4).

Isolated DNA from the animal 42 culture, 30.7 ng/μL, was sequenced using next-generation sequencing producing complete coverage of the ASFV genome. After trimming, 166,165,472 reads remained in pairs for alignment to reference genome. The total number of mapped reads was 6,484,462 with an average coverage of 3303 reads and a minimum coverage of 11 reads. When aligned to the reference 2007 ASFV Georgia sequence, three consensus nucleotide changes were detected. Two mutations were silent, a deletion at residue 19,792 and an A to G mutation at residue 98,378, while a third mutation, C to G at residue 167,188, resulted in an amino acid change in the E199L coding region.

## 4. Discussion

Buffy coat cells extracted from swine blood and treated with media containing an L929 component were an effective way to culture ASFV. While untreated buffy coat cells can produce sparse rosettes following infection, the treatment of these cells with an induction media resulted in dramatic increases in observed rosettes (Figure 1A). This increase in rosette abundance is accompanied by an increase over time in viral genome abundance, as determined by real time PCR, following infection (Figure 1B).

The methodology described herein has practical benefits. Small volumes of blood can be treated either with EDTA or heparin as an anticoagulant and stored at 4°C overnight prior to isolation. Once isolated, cells can be cryopreserved in a basic cryopreservation medium, 90% FBS and 10% DMSO, for an extended period prior to usage. These benefits are especially critical to labs with limited resources as the small blood volumes allow for propagation and sequencing of ASFV without the need for dedicated bleeder pigs and the more invasive isolation of PBMCs or PAMs.

When cultured in M∅ base media, cells of the buffy coat fraction sustained replication of ASFV over a broad range of dilutions from both stock virus (Table 1) and infected swine blood (Table 2). Very small amounts of viable virus are needed for viral replication with replication observed in one of eight wells using stock virus diluted to 10^−7^ from a 10^6.8^ TCID50 stock virus. This was an effective means to culture ASFV from blood of infected animals for use in viral genome sequencing. Culturing samples on buffy coat cells provided positive real time PCR results at higher dilutions than that observed with diluted blood alone (Table 2). This should not be interpreted to say this method is more sensitive than real time PCR for detection but rather a demonstration of how little ASFV is needed to induce replication in susceptible cells.

The ability of multiple ASFV isolates to propagate on buffy coat cells over a broad range of virus levels suggests this methodology could be used for potential pooled blood surveillance at meat harvesting facilities. Such surveillance would be helpful in identifying emerging strains in endemic areas with less virulent circulating ASFV populations [32–35] providing an easy means to detect, isolate, and propagate ASFV for sequencing.

Sequencing new and emerging isolates of ASFV is critical to understanding disease ecology providing insights into virus incursions and identification of less virulent circulating ASFV strains [13, 14, 34, 35]. Blood samples from ASFV-experimentally infected swine produced rosettes over a broad range of dilutions (Table 3). Buffy coat cells provided an effective means to propagate ASFV from the blood of infected swine, even after prolonged storage at 4°C, as all samples produced ASFV DNA (Table 4). Extracted viral DNA contained full genome coverage, and sequencing identified genomic changes relative to the reference sequence.

## 5. Conclusion

Buffy coat cells provide an alternative to standard primary cells, such as PBMCs or PAMs, and provide a practical means to culture ASFV from infected blood for full genome sequencing. The low volumes of blood needed for isolation of buffy coat cells may provide an ideal means to survey and understand virus dynamics within cells of captively held wildlife, particularly those that are currently under threat or endangered. As ASF spreads into non-endemic regions, understanding the susceptibility of *Suidae* beyond domestic swine and warthogs becomes critical for disease ecology. ASFV's entry into south-east Asia had a dramatic effect on local populations of wild and domestic *Suidae* [36]. In the Americas, populations of peccaries, members of the family *Tayassuidae*, are known to contract swine diseases, such as classical swine fever [37]. Previous work showed no evidence of ASFV infection in peccaries upon challenge [37]. This work was validated recently utilizing PBMCs from zoological specimens with recent ASFV outbreak strains [38]. It remains unknown how attenuated ASFV vaccine strains may behave if introduced to wildlife populations.

The usage of primary cell lines is critical for ASFV research beyond research in vaccine development and understanding of virus replication within the cell. Understanding the persistence of ASFV in carcasses and the environment is critical for outbreak recovery. ASFV DNA persists in the environment longer than viable virus [39, 40], making hemadsorption assays on primary cells critical for evaluating virus viability. Buffy coat cells may provide an economic alternative for environmental studies, reducing the required demand of PBMCs [41, 42] or PAMs [43].

## Figures and Tables

**Table 1:** Average *C*_т_ values as determined by real time PCR for ASFV DNA and standard deviation for dilutions of stock ASFV on cells of the buffy coat fraction.

Dilution	1 dpi	5 dpi	7 dpi	14 dpi
10^−2^	26.0 ± 0.4	23.8 ± 1.8	23.9 ± 0.5	14.5⁣^*a*^± 8.0
10^−3^	29.4 ± 0.4	24.3 ± 1.3	22.8 ± 0.4	20.8 ± 4.2
10^−4^	33.3 ± 1.1	26.7 ± 1.2	23.3 ± 0.5	18.9 ± 1.9
10^−5^	38.0⁣^*a*^± 2.3	30.1 ± 3.8	24.2 ± 2.2	18.9 ± 2.5
10^−6^	38.9⁣^*a*^± 0.5	38.2⁣^*a*^	30.6⁣^*a*^± 7.0	17.9⁣^*a*^± 0.6
10^−7^	Undetected	40.1⁣^*a*^	32.0⁣^*a*^	17.4⁣^*a*^
10^−8^	36.9⁣^*a*^	Undetected	Undetected	Undetected

⁣^*a*^Represents not all inoculated wells had detectable ASFV DNA. Standard deviations were not calculated if less than three real time PCR positive wells were present. *N* = 8 samples/day postinfection (dpi).

**Table 2:** Average *C*_т_ as determined by real time PCR for ASFV DNA and standard deviation for dilutions of blood from ASFV-infected swine both independently and cultured on buffy coat fraction cells.

Dilution	Original blood sample	1 dpi	5 dpi	7 dpi	11 dpi	14 dpi
10^−2^	26.1 ± 0.3	22.6 ± 0.5	23.3 ± 2.6	24.4 ± 0.7	27.5⁣^*a*^ ± 5.9	25.7⁣^*a*^± 4.0
10^−3^	28.8 ± 0.2	26.2 ± 0.5	23.2 ± 2.3	23.5 ± 0.8	21.8 ± 2.3	19.8 ± 0.6
10^−4^	32.5 ± 1.5	29.3 ± 0.7	23.3 ± 2.6	22.8 ± 0.6	21.3 ± 2.2	19.2 ± 0.5
10^−5^	37.7 ± 0.3	32.5 ± 1.0	23.7 ± 2.0	22.8 ± 0.4	21.2 ± 1.9	18.9 ± 0.5
10^−6^	41.4⁣^*a*^	36.0 ± 1.7	25.9 ± 0.4	23.0 ± 0.3	21.0 ± 1.8	18.5 ± 0.3
10^−7^	Undetected	37.3 ± 0.5	27.5 ± 1.4	23.6 ± 1.9	20.8 ± 1.5	18.3 ± 0.3
10^−8^	Undetected	37.8⁣^*a*^	42.1⁣^*a*^	34.3⁣^*a*^± 7.2	22.3⁣^*a*^± 2.2	18.3⁣^*a*^± 0.5
10^−9^	Undetected	Undetected	Undetected	Undetected	Undetected	Undetected
10^−10^	Undetected	Undetected	Undetected	38.3⁣^*a*^	25.0⁣^*a*^	19.4⁣^*a*^

⁣^*a*^Represents not all inoculated wells had detectable ASFV DNA. Standard deviations were not calculated if less than three real time PCR positive wells were present. *N* = 8 samples/day postinfection (dpi).

**Table 3:** Blood from seven pigs experimentally infected with ASFV Georgia 2007 was applied in 10-fold serial dilutions on buffy coat cells in M∅ base media and scored for rosettes at 4 and 6 dpi (*n* = 3).

Animal ID	Storage length (days)	Blood dilution						
		10^−2^	10^−3^	10^−4^	10^−5^	10^−6^	10^−7^	
P19-05	1206	3	3	3	3	1	0	
P19-04	1206	3	3	3	3	1	0	
P19-03	1206	3	3	3	3	1	0	
42	660	3	3	3	3	3	1	
41	657	3	3	3	3	0	0	
40	595	3	3	3	2	1	0	
39	595	3	3	3	3	0	0	

**Table 4:** Source swine identification number, average *C*_т_ as determined by real time PCR quantification for ASFV DNA, and concentration of double stranded DNA from ASFV positive blood inoculated buffy coat fraction cell cultures.

Swine ID	Scale of culture	*C*_т_ mean	dsDNA (ng/μL)
42	6-well plate	19.1	30.7
P19-03	6-well plate	18.9	15.6
39	6-well plate	17.5	14.2
P19-05	6-well plate	17.5	6.19
38	6-well plate	29.3	7.56
P19-04	6-well plate	17.2	36.6
38	T-50 flask	25.9	10.9

## Data Availability

All data presented in this manuscript are available from the authors upon reasonable request.

## Supporting Information

Supporting Information Data utilized for Figures 1B, 2A, Tables 1, and 2 are available in online supporting data as Tables S1, S2, S3, and S4, respectively.

## References

[1] R. Eustace Montgomery (1921). On A Form of Swine Fever Occurring in British East Africa (Kenya Colony). *Journal of Comparative Pathology and Therapeutics*.

[2] Rowlands R. J., Michaud V., Heath L. (2008). African Swine Fever Virus Isolate, Georgia 2007. *Emerging Infectious Diseases*.

[3] Gonzales W., Moreno C., Duran U. (2021). African Swine Fever in the Dominican Republic. *Transboundary and Emerging Diseases*.

[4] Sauter-Louis C., Forth J. H., Probst C. (2021). Joining the Club: First Detection of African Swine Fever in Wild Boar in Germany. *Transboundary and Emerging Diseases*.

[5] Maciulskis P., Masiulis M., Pridotkas G. (2020). The African Swine Fever Epidemic in Wild Boar (*Sus scrofa*) in Lithuania (2014–2018. *Veterinary Sciences*.

[6] Mazur-Panasiuk N., Woźniakowski G., Niemczuk K. (2019). The First Complete Genomic Sequences of African Swine Fever Virus Isolated in Poland. *Scientific Reports*.

[7] Zhou X., Li N., Luo Y. (2018). Emergence of African Swine Fever in China, 2018. *Transboundary and Emerging Diseases*.

[8] Woonwong Y., Do Tien D., Thanawongnuwech R. (2020). The Future of the Pig Industry After the Introduction of African Swine Fever Into Asia. *Animal Frontiers*.

[9] Flannery J., Moore R., Marsella L. (2020). Towards a Sampling Rationale for African Swine Fever Virus Detection in Pork Products. *Foods*.

[10] Wang W. H., Lin C. Y., Chang Ishcol M. R. (2019). Detection of African Swine Fever Virus in Pork Products Brought to Taiwan by Travellers. *Emerging Microbes & Infections*.

[11] Kim H. J., Lee M. J., Lee S. K. (2019). African Swine Fever Virus in Pork Brought Into South Korea by Travelers From China, August 2018. *Emerging Infectious Diseases*.

[12] Jurado C., Mur L., Perez Aguirreburualde M. S. (2019). Risk of African Swine Fever Virus Introduction Into the United States Through Smuggling of Pork in Air Passenger Luggage. *Scientific Reports*.

[13] Lakin S. M., O’Donnell V. K., Xu L. (2022). Whole Genome Sequencing and Molecular Epidemiology of the 2021 African Swine Fever Virus Outbreak in the Dominican Republic. *Transboundary and Emerging Diseases*.

[14] Zhenzhong W., Chuanxiang Q., Shengqiang G. (2022). Genetic Variation and Evolution of Attenuated African Swine Fever Virus Strain Isolated in the Field: A Review. *Virus Research*.

[15] Bisimwa P. N., Ongus J. R., Steinaa L. (2021). The First Complete Genome Sequence of the African Swine Fever Virus Genotype X and Serogroup 7 Isolated in Domestic Pigs From the Democratic Republic of Congo. *Virology Journal*.

[16] Portugal R., Goatley L. C., Husmann R., Zuckermann F. A., Dixon L. K. (2020). A Porcine Macrophage Cell Line that Supports High Levels of Replication of OURT88/3, an Attenuated Strain of African Swine Fever Virus. *Emerging Microbes & Infections*.

[17] Kameyama K. I., Kitamura T., Okadera K., Ikezawa M., Masujin K., Kokuho T. (2022). Usability of Immortalized Porcine Kidney Macrophage Cultures for the Isolation of ASFV Without Affecting Virulence. *Viruses*.

[18] Genovesi E. V., Knudsen R. C., Gerstner D. J. (1989). In Vitro Induction of Swine Peripheral Blood Monocyte Proliferation by the Fibroblast-Derived Murine Hematopoietic Growth Factor CSF-1. *Veterinary Immunology and Immunopathology*.

[19] Martins C. L., Scholl T., Mebus C. A., Fisch H., Lawman M. J. (1987). Modulation of Porcine Peripheral Blood-Derived Macrophage Functions by in Vitro Infection With African Swine Fever Virus (ASFV) Isolates of Different Virulence. *Viral Immunology*.

[20] Genovesi E. V., Villinger F., Gerstner D. J., Whyard T. C., Knudsen R. C. (1990). Effect of Macrophage-Specific Colony-Stimulating Factor (CSF-1) on Swine Monocyte/Macrophage Susceptibility to in Vitro Infection by African Swine Fever Virus. *Veterinary Microbiology*.

[21] Carrascosa A. L., Santarén J. F., Viñuela E. (1982). Production and Titration of African Swine Fever Virus in Porcine Alveolar Macrophages. *Journal of Virological Methods*.

[22] Forman A. J., Wardley R. C., Norley S. G. (1983). Interactions of Porcine Alveolar Macrophages and Bone Marrow Cells With African Swine Fever Virus and Virus-Infected Cells. *Veterinary Microbiology*.

[23] Karalova E. M., Sargsyan Kh V., Hampikian G. K. (2011). Phenotypic and Cytologic Studies of Lymphoid Cells and Monocytes in Primary Culture of Porcine Bone Marrow During Infection of African Swine Fever Virus. *In Vitro Cellular & Developmental Biology - Animal*.

[24] Malmquist W. A., Hay D. (1960). Hemadsorption and Cytopathic Effect Produced by African Swine Fever Virus in Swine Bone Marrow and Buffy Coat Cultures. *American Journal of Veterinary Research*.

[25] Denham S., Brookes S. M., Hutchings G. H., Parkhouse R. M. (1996). Granulocyte-Macrophage Colony Stimulating Factor Promotes Prolonged Survival and the Support of Virulent Infection by African Swine Fever Virus of Macrophages Generated From Porcine Bone Marrow and Blood. *Journal of General Virology*.

[26] Wardley R. C., Wilkinson P. J. (1978). The Growth of Virulent African Swine Fever Virus in Pig Monocytes and Macrophages. *Journal of General Virology*.

[27] Englen M. D., Valdez Y. E., Lehnert N. M., Lehnert B. E. (1995). Granulocyte/Macrophage Colony-Stimulating Factor Is Expressed and Secreted in Cultures of Murine L929 Cells. *Journal of Immunological Methods*.

[28] Stanley E. R. (1985). The Macrophage Colony-Stimulating Factor, CSF-1. *Methods in Enzymology*.

[29] Mayer P. (1983). The Growth of Swine Bone Marrow Cells in the Presence of Heterologous Colony Stimulating Factor: Characterization of the Developing Cell Population. *Comparative Immunology, Microbiology and Infectious Diseases*.

[30] Zsak L., Borca M. V., Risatti G. R. (2005). Preclinical Diagnosis of African Swine Fever in Contact-Exposed Swine by a Real-Time PCR Assay. *Journal of Clinical Microbiology*.

[31] Spinard E., Dinhobl M., Erdelyan C. N. G. (2024). A Standardized Pipeline for Assembly and Annotation of African Swine Fever Virus Genome. *Viruses*.

[32] Njau E. P., Machuka E. M., Cleaveland S. (2021). African Swine Fever Virus (ASFV): Biology, Genomics and Genotypes Circulating in Sub-Saharan Africa. *Viruses*.

[33] Arias M., Jurado C., Gallardo C., Fernandez-Pinero J., Sanchez-Vizcaino J. M. (2018). Gaps in African Swine Fever: Analysis and Priorities. *Transboundary and Emerging Diseases*.

[34] Gallardo C., Soler A., Nieto R. (2015). Experimental Transmission of African Swine Fever (ASF) Low Virulent Isolate NH/P68 by Surviving Pigs. *Transboundary and Emerging Diseases*.

[35] Owolodun O. A., Obishakin E. T., Ekong P. S., Yakubu B. (2010). Investigation of African Swine Fever in Slaughtered Pigs, Plateau State, Nigeria, 2004–2006. *Tropical Animal Health and Production*.

[36] Ewers R. M., Nathan S., Lee P. A. K. (2021). African Swine Fever Ravaging Borneo’s Wild Pigs. *Nature*.

[37] Dardiri A. H., Yedloutschnig R. J., Taylor W. D.

[38] Friedrichs V., Deutschmann P., Carrau T. (2025). Investigating African Swine Fever Virus Susceptibility Across Seven Genera of Peccaries and Pigs Using Peripheral Blood Mononuclear Cells. *Journal of Zoo and Aquarium Research*.

[39] Mazur-Panasiuk N., Wozniakowski G. (2020). Natural Inactivation of African Swine Fever Virus in Tissues: Influence of Temperature and Environmental Conditions on Virus Survival. *Veterinary Microbiology*.

[40] Prodelalova J., Kavanova L., Salat J. (2022). Experimental Evidence of the Long-Term Survival of Infective African Swine Fever Virus Strain Ba71V in Soil Under Different Conditions. *Pathogens*.

[41] Zani L., Masiulis M., Busauskas P. (2020). African Swine Fever Virus Survival in Buried Wild Boar Carcasses. *Transboundary and Emerging Diseases*.

[42] Carlson J., Fischer M., Zani L. (2020). Stability of African Swine Fever Virus in Soil and Options to Mitigate the Potential Transmission Risk. *Pathogens*.

[43] Stoian A. M. M., Zimmerman J., Ji J. (2019). Half-Life of African Swine Fever Virus in Shipped Feed. *Emerging Infectious Diseases*.

